# Behavior of AV synchrony pacing mode in a leadless pacemaker during variable AV conduction and arrhythmias

**DOI:** 10.1111/jce.15061

**Published:** 2021-05-20

**Authors:** Christophe Garweg, Surinder Kaur Khelae, Joseph Yat Sun Chan, Larry Chinitz, Philippe Ritter, Jens Brock Johansen, Venkata Sagi, Laurence M. Epstein, Jonathan P. Piccini, Mario Pascual, Lluis Mont, Rik Willems, Vincent Splett, Kurt Stromberg, Todd Sheldon, Nina Kristiansen, Clemens Steinwender

**Affiliations:** ^1^ Department of Cardiovascular Sciences University Hospitals Leuven, University of Leuven Leuven Belgium; ^2^ Department of Electrophysiology Institut Jantung Negara Kuala Lumpur Malaysia; ^3^ Department of Medicine and Therapeutics, Prince of Wales Hospital Chinese University of Hong Kong Shatin Hong Kong; ^4^ Leon H. Charney Division of Cardiology NYU Langone Medical Center New York New York USA; ^5^ Department of Electrophysiology and Cardiac Stimulation Hôpital Haut‐ Lévêque–CHU de Bordeaux Pessac France; ^6^ Department of Cardiology Odense University Hospital Odense Denmark; ^7^ Baptist Heart Specialists Baptist Medical Center Jacksonville Florida USA; ^8^ Department of Electrophysiology North Shore University Hospital Manhasset New York USA; ^9^ Division of Cardiology Duke University Medical Center and Duke Clinical Research Institute Durham North Carolina USA; ^10^ Miami Cardiac & Vascular Institute Baptist Hospital Miami Florida USA; ^11^ Institut Clinic Cardiovascular (ICCV), Hospital Clínic Universitat de Barcelona Barcelona Catalonia Spain; ^12^ Medtronic, Inc. Minneapolis Minnesota USA; ^13^ Medtronic Bakken Research Center Maastricht The Netherlands; ^14^ Department of Cardiology, Kepler University Hospital, Medical Faculty Johannes Kepler University Linz Austria; ^15^ Department of Cardiology Paracelsus Medical University Salzburg Salzburg Austria

**Keywords:** arrhythmias, atrial fibrillation, AV synchrony, leadless pacing, paroxysmal AV block

## Abstract

**Introduction:**

MARVEL 2 assessed the efficacy of mechanical atrial sensing by a ventricular leadless pacemaker, enabling a VDD pacing mode. The behavior of the enhanced MARVEL 2 algorithm during variable atrio‐ventricular conduction (AVC) and/or arrhythmias has not been characterized and is the focus of this study.

**Methods:**

Of the 75 patients enrolled in the MARVEL 2 study, 73 had a rhythm assessment and were included in the analysis. The enhanced MARVEL 2 algorithm included a mode‐switching algorithm that automatically switches between VDD and ventricular only antibradycardia pacing (VVI)‐40 depending upon AVC status.

**Results:**

Forty‐two patients (58%) had persistent third degree AV block (AVB), 18 (25%) had 1:1 AVC, 5 (7%) had variable AVC status, and 8 (11%) had atrial arrhythmias. Among the 42 patients with persistent third degree AVB, the median ventricular pacing (VP) percentage was 99.9% compared to 0.2% among those with 1:1 AVC. As AVC status changed, the algorithm switched to VDD when the ventricular rate dropped less than 40 bpm. During atrial fibrillation (AF) with ventricular response greater than 40 bpm, VVI‐40 mode was maintained. No pauses longer than 1500 ms were observed. Frequent ventricular premature beats reduced the percentage of AV synchrony. During AF, the atrial signal was of low amplitude and there was infrequent sensing.

**Conclusion:**

The mode switching algorithm reduced VP in patients with 1:1 AVC and appropriately switched to VDD during AV block. No pacing safety issues were observed during arrhythmias.

## 1 INTRODUCTION

The prevalence of heart conduction disorders requiring implantation of a permanent pacemaker is increasing as the population is aging. Nevertheless, despite all technological improvements over the last 6 decades, conventional pacing therapy is still associated with a complications rate ranging from 1% to 12.4% in the first 2‐month post implantation and reported up to 19.7% at 5‐year follow‐up.^1^, ^2^, ^3^

Leadless cardiac pacing has demonstrated an excellent safety and efficacy profile combining a high implant success rate (99.1%), a low major complication rate through 12 months (2.7%), and stable electrical parameters over time.^5^ Currently, leadless pacing is considered a safe alternative to conventional pacing systems if asynchronous ventricular pacing (VVI pacing mode) is acceptable and may also be chosen based upon a patient's condition (elderly, co‐morbidities, high risk for complications). Therefore, implantation of a single‐chamber ventricular pacing device is limited to 14%–32% of all pacemaker implants as the maintenance of atrio‐ventricular synchrony (AVS) is preferred in patients with high degree AV block and preserved sinus rhythm.^5^, ^6^

Recently, the Micra atrial tracking using a ventricular accelerometer (MARVEL) 2 study demonstrated high AV synchronous pacing at rest (mean 89.2%) in patients previously implanted with a ventricular leadless pacemaker and presenting with third degree AV block and normal sinus rhythm between 60 and 100 beats per minute.^8^

Even if AV synchrony is maintained, a high percentage of right ventricular pacing can have deleterious effects, including increased risk of developing atrial fibrillation (AF), left ventricular dysfunction, heart failure, and death.^8^, ^9^ Therefore, in patients with paroxysmal high degree AV block or slightly prolonged AV conduction (AVC), intrinsic AVC should be promoted to prevent unnecessary right ventricular pacing.

In addition, the occurrence of paroxysmal or persistent AF is not infrequent (ranging from 3% to 39.9%) during follow‐up even if there is no history of atrial arrhythmias at the time of the implantation procedure.^10^, ^11^, ^12^ Limited data from the MARVEL Evolve study indicated that the algorithm behaved safely during AF, as the arrhythmia was not tracked due to the absence of efficient mechanical atrial activity.^14^

In this analysis of the MARVEL 2 study, we report the behavior of the MARVEL 2 algorithm in patients with AVC status other than persistent third degree AV block with normal sinus rhythm and in the presence of sinus arrhythmia, sinus bradycardia (<40 bpm), or atrial/ventricular premature beats and atrial arrhythmias.

## 2 METHODS

### 2.1 Study design

The MARVEL 2 study was a prospective, non‐randomized multi‐center clinical trial.^8^ The primary aim of the MARVEL 2 study was to confirm the ability of a downloaded algorithm (hereafter referred to as the MARVEL 2 algorithm) to provide AV synchronous pacing by mechanically sensing atrial contraction via the accelerometer signal (VDD pacing) from a Micra leadless pacemaker (Medtronic, Inc.) implanted in the right ventricle. The primary efficacy objective was to demonstrate the superiority of the MARVEL 2 algorithm to provide AV synchronous pacing relative to VVI pacing in subjects with persistent third degree AV block and normal sinus node function at rest.

The protocol was approved by all local ethics committees and national regulatory agencies at each participating institution. All patients provided written informed consent.

### 2.2 Patients and procedures

The 75 subjects enrolled in the MARVEL 2 study who received the software download were eligible for inclusion in this analysis. These patients were older than 18 years, had a history of AV block, and were previously implanted or undergoing implant with a Micra leadless pacemaker.

The MARVEL 2 study procedures have been previously described.^8^ Briefly, custom software was temporarily downloaded in the Micra. A specialized Holter monitor was placed on the patient during study procedures to collect electrocardiograph (ECG), electrogram, accelerometer waveform, and device markers. Initial algorithm parameter settings were set by an automatic setup algorithm during 20 min of VVI‐50 pacing. The performance of the AV synchronous pacing mode and associated mode switches was characterized over the entire study duration which averaged 153 ± 29 min.

### 2.3 Echocardiographic analysis

Echocardiograms were collected from each patient during VVI and VDD pacing following a standard protocol. Since patients with 1:1 AVC would have their intrinsic rhythm during both VVI and VDD mode (due to the AVC mode switch), these patients were programmed to VVI pacing with a lower rate 5–10 bpm above their intrinsic rate during VVI mode. An echocardiography core laboratory (United Heart and Vascular Clinic, St. Paul, Minnesota), blinded to patient and pacing mode, measured left ventricular outflow (LVOT) velocity‐time integral (VTI) during six cardiac cycles in each pacing mode.

### 2.4 Atrial sensing algorithm

The algorithm incorporates a post‐ventricular blanking period and a dual threshold detection method. The first threshold (A3 threshold) that occurs early is used for detecting the atrial contraction when A4 (atrial kick) occurs during the A3 (passive filling) time and is less sensitive. The second threshold (A4 threshold) occurs later in the cycle after A3 has occurred and is more sensitive. Because of the importance of the A3 sensing window, a telemetry marker (VE) is displayed at the end of the A3 window (Figure S1).

### 2.5 AV conduction (AVC) mode switch

The AVC mode switch is designed to promote intrinsic AVC during periods of 1:1 AVC in patients with paroxysmal AV block. Periodic conduction checks are performed by switching the pacing mode from VDD to VVI with a lower rate of 40 bpm (VVI‐40) for at least two beats. If AVC is present, the device remains in VVI mode with a lower rate of 40 bpm. If at any time AVC is lost, as indicated by at least two of four beats being paced, the device switches back to VDD mode. The first check for AVC occurs after 1 min. Subsequent checks occur at progressively longer intervals (2, 4, 8 … min) up to 8 h and then occur every 8 h thereafter.

### 2.6 Rate smoothing algorithm

The algorithm incorporates a rate smoothing feature to maintain AVS during intermittent A4 undersensing in the presence of a relatively stable sinus rate. If an atrial contraction (A4) is not detected, a ventricular pace is delivered at a programmable rate smoothed interval (typically 100 ms) longer than the recent R‐R intervals. Following a ventricular sensed beat, such as a premature ventricular contraction (PVC), the lower rate is the programmed lower rate, not the rate smoothing interval.

## 3 STATISTICAL ANALYSIS

Each patient's predominant heart rhythm was determined as persistent 3rd degree AV block, intact AVC, or other (e.g., atrial arrhythmias or other AVC status) based on P‐R intervals during automatic setup phase and P‐P intervals during both the automatic setup and resting phases. Continuous variables are presented as mean ± *SD* or median and interquartile range. Categorical variables are presented as counts and percentages. LVOT VTI during VVI pacing and while the MARVEL 2 algorithm features were enabled were compared using paired *t* tests. The Wilcoxon signed‐rank test was used to compare ventricular pacing percentage between VVI lower rate pacing and while the MARVEL 2 features were enabled. Statistical analyses were performed using SAS v9.4 (Cary) or R (www.r-project.org).

## 4 RESULTS

### 4.1 Patients

Overall, 75 patients were enrolled in the MARVEL 2 study and received the software download. The average age was 77.5 ± 11.8 years (range 21–94), 30 (40%) were female, and patients had been implanted with a Micra for a median of 9.7 months (range 0–62.1; interquartile range [IQR]: 2.1–18.8). Two of the enrolled subjects were not included in the analyses, since an assessment of the predominant rhythm was not possible due to noise on the ECG. Patient characteristics are summarized in Table 1. Of the 73 patients with useable datasets, during study procedures, 42 (58%) had persistent third degree AV block, 18 (25%) had 1:1 AVC, 5 (7%) had variable AVC, and 8 (11%) had AF or atrial flutter. In contrast to the original MARVEL 2 study, we included two patients with sinus arrhythmias in the third degree AV block cohort, since those arrhythmias do not affect the performance of the AV conduction mode switch. One of the patients with third degree AV block and five of the subjects with 1:1 AVC had sinus bradycardia. Of the five patients with varying AVC, two had intermittent third degree AV block, two had second degree AV block, and one had alternating second and third degree AV block during the study. Two patients with persistent third degree AV block had the AVC mode switch feature disabled to promote atrial tracking due to a ventricular/junctional escape rhythm greater than 40 bpm (example shown in Figure S2). There were no pacing‐related adverse events reported during the study regardless of underlying rhythm.

**Table 1:** Patient baseline characteristics

**Patient characteristics**	**Enrolled** a **(*N* = 75)**	**AV Block (*N* = 42)**	**1:1 AVC (*N* = 18)**	**Other rhythms (*N* = 13)**
Age (years)				
Mean ± standard deviation	77.5 ± 11.8	76.5 ± 13.0	76.4 ± 12.0	82.3 ± 6.8
Median	81.0	80.0	80.5	84.0
25th–75th Percentile	72.0–85.0	70.0–84.0	71.0–84.0	78.0–87.0
Minimum–maximum	21.0–94.0	21.0–94.0	39.0–88.0	69.0–90.0
Female	30 (40.0%)	22 (52.4%)	7 (38.9%)	1 (7.7%)
BMI				
Mean ± standard deviation	26.2 ± 5.7	27.2 ± 6.2	25.9 ± 5.8	23.8 ± 3.6
Median	25.4	25.6	25.6	23.3
25th–75th Percentile	22.6–28.0	23.5–28.7	23.6–27.2	21.3–25.0
Minimum–maximum	17.3–49.2	20.4–49.2	17.3–45.8	18.9–30.8
LVEF				
Mean ± standard deviation	53.5 ± 3.8	53.6 ± 3.9	54.6 ± 3.0	52.6 ± 4.2
Median	54.0	54.0	55.0	53.0
25th–75th Percentile	52.0–56.0	52.0–56.0	54.0–56.0	52.0–55.0
Minimum–maximum	40.0–61.0	40.0–60.0	48.0–61.0	42.0–58.0
RVEF				
Mean ± standard deviation	43.0 ± 8.2	42.7 ± 9.3	44.6 ± 6.2	43.1 ± 6.9
Median	42.9	42.7	43.7	42.1
25th–75th Percentile	37.3–47.5	35.6–50.0	40.3–46.5	40.3–46.1
Minimum–maximum	21.3–62.0	21.3–59.0	34.9–62.0	33.5–57.6
LA end diastolic volume (ml)				
Mean ± standard deviation	54.9 ± 19.4	51.7 ± 18.0	54.2 ± 20.1	63.0 ± 20.4
Median	56.0	47.0	58.0	67.0
25th Percentile‐75th Percentile	39.0–68.0	36.0–65.0	33.0–67.0	52.0–74.0
Minimum–maximum	19.0–94.0	27.0–92.0	22.0–88.0	19.0–90.0
Months from Micra Implant				
Mean ± standard deviation	13.8 ± 14.6	14.6 ± 16.4	13.7 ± 14.3	11.8 ± 10.0
Median	9.7	9.3	9.1	13.5
25th–75th Percentile	2.1–18.8	0.4–21.5	4.4–22.3	2.1–18.1
Minimum–maximum	0.0–62.1	0.0–62.1	0.0–55.7	0.0–31.9
Comorbidities				
Hypertension	52 (69.3%)	29 (69.0%)	12 (66.7%)	10 (76.9%)
Atrial fibrillation	14 (18.7%)	3 (7.1%)	3 (16.7%)	8 (61.5%)
Diabetes	13 (17.3%)	7 (16.7%)	4 (22.2%)	2 (15.4%)
Coronary artery disease	23 (30.7%)	9 (21.4%)	9 (50.0%)	5 (38.5%)
COPD	7 (9.3%)	4 (9.5%)	2 (11.1%)	1 (7.7%)
Dialysis	3 (4.0%)	1 (2.4%)	2 (11.1%)	0 (0.0%)

Abbreviations: AV, atrio‐ventricular; AVC, atrio‐ventricular conduction; BMI, body mass index; COPD, chronic obstructive pulmonary disease; ECG, electrocardiography; LA, left atrium; LVEF, left ventricular ejection fraction; RVEF, right ventricular ejection fraction.

^a^ Two subjects were enrolled, however due to noise on the ECG and low‐amplitude P‐waves, an assessment of the predominant rhythm was not possible, and these two subjects are not included in the analyses.

### 4.2 AV conduction mode switch

**Figure 1:**
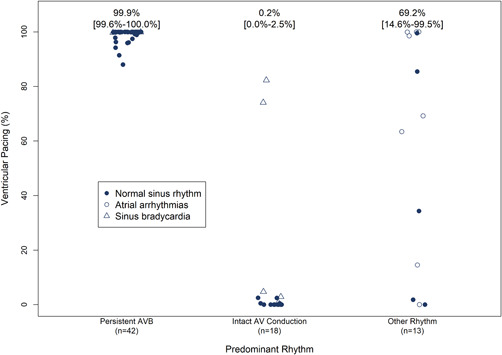
Percentage ventricular pacing by rhythm. The two subjects with sinus bradycardia and high percentage pacing had sinus rate frequently less than 40 bpm, leading to ventricular pacing at lower rate (and mode switching to VDD mode). Percentage pacing varied during atrial arrhythmias depending upon conduction of the AF to the ventricles. Values represent median and interquartile range (in brackets). AF, atrial fibrillation; AVB, complete (third degree) AV block; NSF, normal sinus node function

**Figure 2:**
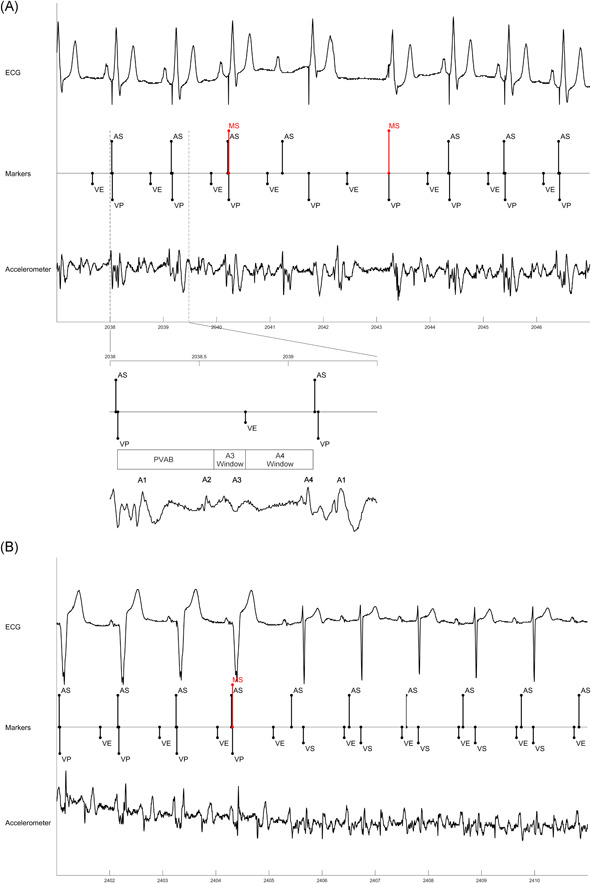
(A) AV conduction mode switch in patient with third degree heart block. Initial mode switch to VVI‐40 does not find 1:1 AV conduction so pacing mode switches to VDD. Lower waveform shows accelerometer signal expanded out for one beat to show individual components of cardiac cycle and blanking (PVAB) and sensing (A3 Window and A4 Window) periods. (B) AV conduction mode switch in patient with 1:1 AV conduction (and first degree AVB). Mode switch to VVI‐40 finds 1:1 AV conduction, so mode remains VVI‐40. AS, atrial sense; MS, mode switch; VE, end of A3 (ventricular) window; VP, ventricular pace; VVI, ventricular only antibradycardia pacing

Among the subjects with persistent third degree AV block the median percentage of ventricular pacing was 99.9% (IQR: 99.6%–100%) compared to 0.2% (IQR: 0.0%–2.5%) among those with 1:1 AVC (Figure 1). A high percentage of ventricular pacing (82.3% and 74.1%) was observed in two patients with 1:1 AVC due to intermittent sinus bradycardia at a sinus rate below 40 bpm. In patients with variable AVC, the algorithm appropriately switched to VDD when less than 2 of 4 ventricular beats were sensed (Table 2). One patient with 1:1 AVC, a PR interval greater than 300 ms, and percentage of ventricular pacing less than 1% was not classified as AVS, since AVS was defined as a PR interval of less than 300 ms. During periods of 1:1 AVC or atrial fibrillation with a ventricular response greater than 40 bpm, VVI‐40 mode was maintained. An example of an AVC check in a patient with persistent third degree AV block mode with a return to VDD pacing mode is shown in Figure 2a and an AVC mode switch to VVI‐40 in a patient with 1:1 AVC is shown in Figure 2b.

**Table 2:** AV synchrony in patients with variable AV conduction

**Patient #**	**AV conduction**	**% Time in VDD mode (rest)**	**AV synchrony (%)**
1	1:1 and third degree AV block	0.0	5.9^a^
2	1:1 and third degree AV block	0.0	100
3	Second degree AV block	44.8	43.4
4	Second and third degree AV block	99.8	98.1
5	Second degree AV block	99.4	95.4

Abbreviations: AV, atrio‐ventricular; VP, ventricular pacing.

^a^ This patient had a long P‐R interval (median, 375 ms) and 1.8% VP. The pre‐defined definition of AV synchrony was P‐R < 300 ms, which led to the low AV synchrony.

Overall, there were 379 AVC checks in the 73 patients during an average Holter recording length of 153 ± 29 min. Among the patients with third degree AV block, there were 210 AVC checks. In all 210 AVC checks, the pacing mode appropriately returned to VDD and no pauses longer than 1500 ms were observed. In the patients with 1:1 AVC (*n* = 18), 16 had a mode switch to VVI‐40. In the other two patients, the mode remained in VDD due to sinus bradycardia less than 40 bpm. In patients with 1:1 AVC and a PR interval greater than 200 ms (*n* = 9), there was always correct mode switching to VVI‐40.

**Figure 3:**
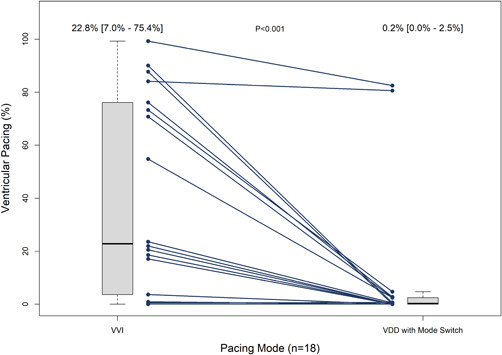
Reduction in percentage of ventricular pacing from VVI (Micra VR initial interrogation) to VDD mode with activated mode switch to VVI‐40. Values above each boxplot represent median and interquartile range. VVI, ventricular only antibradycardia pacing

In the patients with 1:1 AVC, the median ventricular pacing with permanent pacing parameters decreased from a median of 22.8% (IQR: 7.0%–75.4%) during VVI pacing to a median of 0.2% (IQR: 0%–2.5%) during the AVC mode switch (*p* < .001, Figure 3). The ventricular rate during the AVC mode switch was similar to the mean permanent programmed lower rate (59.1 ± 10.6 vs. 56.4 ± 5.9 bpm, *p* = .367). Four patients had a median ventricular rate less than 50 bpm when the AVC mode switch was active. No patients reported symptoms related to the ventricular pacing rate during the study. Stroke volume, measured by LVOT VTI, was higher when 1:1 AVC was promoted by the AVC mode switch compared with RV pacing at slightly above the intrinsic rate (22.7 ± 4.2 vs. 20.4 ± 4.5 cm, *p* = .020).

### 4.3 Atrial fibrillation or flutter

In patients with AF (*n* = 7), the accelerometer signal was of low amplitude and there was infrequent sensing, resulting in either ventricular pacing at the lower rate (Figures S3A,B), or at the conducted rate if AF was intermittently conducted to the ventricles (Figure S3C). In three patients, AF was not conducted to the ventricles. In two other patients, the conduction was greater than 85%, and in the remaining two patients, conduction was approximately 30%. In patients with AF conducted with a ventricular rate greater than 40 bpm, the AVC mode switch switched to VVI‐40 pacing and during intermittent bradycardia, the mode switched back to VDD. There was no ventricular pacing at the upper tracking rate.

In the one patient with atrial flutter, there was still some organized mechanical atrial activity that was recognized by the accelerometer, resulting in atrial tracking at a median ventricular pacing rate of 67 bpm (IQR: 66–67 bpm).

### 4.4 Behavior during premature ventricular contractions (PVCs) and premature atrial contractions (PACs)

**Figure 4:**
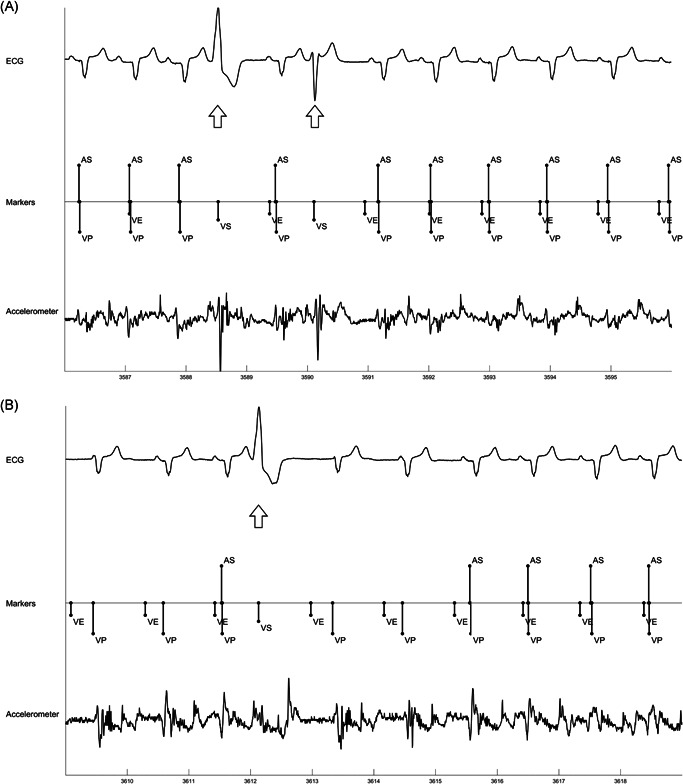
Example behavior during PVCs. (A) shows minimal disruption in atrial sensing after a PVCs (arrow). (B) shows multiple beats are needed to re‐establish AV synchrony since sinus beat occurred after ventricular pace beats. AS, atrial sense; PVC, premature ventricular contraction; VE, end of A3 (ventricular) window; VP, ventricular pace

PVCs disrupt AVS, since atrial contraction typically occurs in ventricular systole (Table 3, Figure 4). Of note, one subject with 1:1 AVC, where 100% AVS would be expected, had an AVS of 63.4% due to a PVC burden of 35.8%. Since rate smoothing is not applied on sensed ventricular beats, including PVCs, the compensatory pause following the PVC allows additional time for atrial sensing and to re‐establish AV synchronization (Figure 4A). However, in some cases, depending upon the relative timing between the PVC and sinus beat, it may take more than one beat to re‐establish AVS (Figure 4B).

**Table 3:** AV synchrony in patients with high PVC burden

**Patient #**	**AV conduction status**	**Sinus node status**	**PVC burden**	**AV synchrony (%)**
1	1:1	Normal	35.8	63.4
2	Third degree AV block	Normal	9.2	81.9
	
3	Intermittent third degree AV block	Normal	8.3	5.9^a^
4	Third degree AV block	Normal+PACs	4.6	92.5
5	Second degree AV block	Sinus brady	5.6	95.4

Abbreviations: AV, atrio‐ventricular; PVC, premature ventricular contraction.

^a^ This patient had a long P‐R interval (median, 375 ms). The pre‐defined definition of AV synchrony was P‐R < 300 ms, which led to the low AV synchrony.

**Figure 5:**
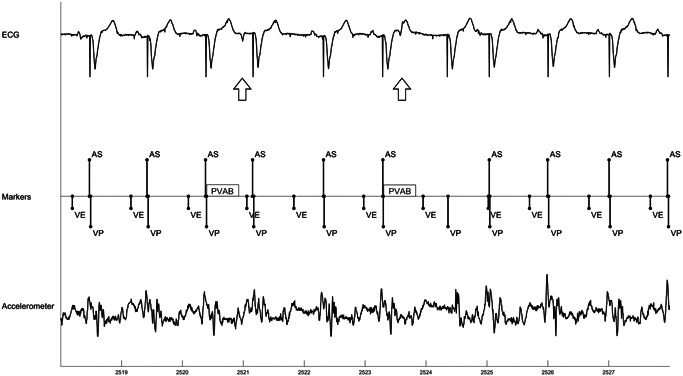
Example behavior during PACs. A late coupled PAC (left arrow) is tracked without loss of AV synchrony while an early coupled PAC (right arrow) is not tracked since it is in the blanking period (PVAB). AV, atrio‐ventricular; PAC, premature atrial contraction; PVC, premature ventricular contraction

The atrial sensing behavior during PACs depends upon the coupling interval of the PACs. If the coupling interval is long, then the PAC may be tracked (example shown in Figure 5), without any loss of AVS. If the coupling interval is short, then the atrial contraction will not be tracked if it occurs during post ventricular atrial blanking, but it could be tracked during the A3 window (i.e., before VE marker) if the PAC contraction combines with the A3 passive filling signal and crosses the A3 threshold.

## 5 SINUS ARRHYTHMIA

In the presence of sinus arrhythmia, rate smoothing may prematurely deliver a ventricular pace, creating temporary loss of AVS (example shown in Figure S4A). Programming a longer rate smoothing delta or turning off rate smoothing, will extend the VP‐VP interval and may allow tracking of atrial contraction.

In contrast, at higher sinus rates with low sinus rate variability, a shorter rate smoothing delta may be preferred. An undersensed A4 coupled with a longer rate smoothing delta can move the subsequent A4 out of the A4 sensing window into ventricular systole (Figure S4B), whereas a shorter value may improve AVS in the presence of intermittent A4 undersensing (Figure S4C).

## 6 DISCUSSION

This study primarily investigated the safety and behavior of the MARVEL 2 algorithm in patients not presenting with persistent third degree AV block and normal sinus node function during the duration of the study and confirmed the safety of the AVC mode switch.

### 6.1 AV conduction mode switch

The AVC mode switch was developed to reduce the percentage of ventricular pacing in patients with intermittent high degree AV block. During the VDD pacing mode, the MARVEL 2 algorithm checks periodically for intrinsic AVC by switching to VVI pacing at a lower rate of 40 bpm. If sensed ventricular events occur at greter than 40 bpm, the device remains in VVI mode (called VVI+ mode). When the VVI+ mode is activated, the atrial sensing is turned off, meaning that patients can present an intrinsic ventricular rhythm greater than 40 bpm without AV synchrony. If ≥2 of 4 consecutive ventricular beats are paced, it reverts to VDD pacing mode. We showed that the algorithm significantly reduced the ventricular pacing percentage in patients with intrinsic ventricular rhythm greater than 40 bpm. This represents three potential advantages for patients. First, it could reduce ventricular pacing induced left ventricular dysfunction. Second, it could reduce atrial fibrillation.^8^, ^9^ Finally, the reduction in ventricular pacing and the ability to place the accelerometer in a standby mode will improve longevity.

The safety of the AVC mode switch was confirmed in patients with persistent third‐degree AV block as it did not induce ventricular pauses greater than 1500 ms, arrhythmias, or symptoms. Future studies will have to confirm the safety of the mode switch in clinical practice.

Based on these different considerations, we recommend evaluating the activation of the AVC mode switch on a patient‐by‐patient basis. For example, physicians should consider deactivating the mode switch in different clinical situations, such as: (1) patients without ventricular escape rhythm, (2) patients with second degree AV block (2:1 AV conduction) and sinus rhythm greater than 80 bpm, or (3) patients with complete AV block and a ventricular escape rhythm greater than 40 bpm. Also, the mode switch operates independently of the AV interval: patients with long first‐degree AV block greater than 300 ms will not be paced. Finally, patients having a sinus rate less than 40 bpm with intrinsic conduction will also not benefit from this mode switch. In these patients, a dual chamber pacing system with atrial pacing is required if AVS is preferred.

### 6.2 Behavior during arrhythmias

Our study also confirmed that the MARVEL 2 algorithm behaved in a safe manner in the presence of different types of arrhythmias. The MARVEL 2 algorithm does not contain a mode switching as contained in the traditional pacemaker with electrical sensing of the atrium but its utility seems low as observed in our population including eight patients presenting with atrial arrhythmias (seven patients with atrial fibrillation and one with atrial flutter). During atrial arrhythmias, the accelerometer signals were of low amplitude. The automatic features of the device will adjust the A4 threshold to increase sensitivity, possibly leading to intermittent oversensing of atrial activity or noise. Nevertheless, no episodes of oversensing induced tachycardia greater than 100 bpm were observed. It is worth noting that intermittent atrial oversensing could also be related to the presence of a reduced intermittent atrial mechanical activity during atrial fibrillation. Previously, Fujii et al.^15^ showed that a large and relatively slow fibrillatory atrial electrical activity could induce a slight atrial contraction with a visible hemodynamic effect during echocardiography.

Second, the occurrence of atrial or ventricular premature beats is not infrequent during patient follow‐up. In the presence of frequent PACs, the behavior of the MARVEL 2 algorithm depends on the timing of the atrial event. If the PAC occurs early in the cardiac cycle after a ventricular event in post‐ventricular atrial blanking, it will not be sensed, preventing any pacemaker induced tachycardia. Conversely, in the presence of a late‐coupled PAC, an A4 signal can be recognized and be followed by a ventricular pace. The presence of frequent PVCs caused a reduction in AVS similar to that observed in conventional dual chamber pacing systems.

Induction of ventricular arrhythmias by pacing facilitated short‐long‐short sequences is a known behavior of pacemaker timing.^15^, ^16^ No ventricular  using a leadless pacing system was evaluated at rest in a limited patient sample for a short duration (the MARVEL 2 software was downloaded for a maximum 5 h) during a single study visit. Behavior of the MARVEL 2 algorithm during the different rhythms and varying AVC was assessed at rest and its performance during activities need to be studied.

## 7 CONCLUSION

The MARVEL 2 algorithm permits a reduction in the percentage of ventricular pacing in the presence of 1:1 AV conduction and sinus rhythm greater than 40 bpm, while it appropriately switches to VDD during episodes of high degree AV block. The algorithm behaved safely during arrhythmias, with no pacing‐related adverse events reported during the study period. These encouraging data should be confirmed by further studies in the real‐world setting.

## DISCLOSURES

Christophe Garweg contributed to research funding, speaker/consultancy fees Medtronic. Surinder Kaur Khelae Speakers Bureau: Bayer/Schering Pharma, Boston Scientific, Medtronic, Pfizer; Joseph Yat Sun Chan: Honoraria: Medtronic; Larry Chinitz contributed to fees for services: Abbott, Biosense Webster, Pfizer, Biotronik, Medtronic. Fellowship support: Biotronik, Boston Scientific, Medtronic. Philippe Ritter contributed to fees for service: Medtronic. Jens Brock Johansen: Speakers Bureau: Medtronic, Merit Medical. Honoraria/Scientific Board: Medtronic, Biotronik. Venkata Sagi h.

## Supporting information

Supporting information.
