# Supplementary material for: Behavior of AV synchrony pacing mode in a leadless pacemaker during variable AV conduction and arrhythmias
Source: J Cardiovasc Electrophysiol. 2021 May 20;32(7):1947–57. doi: 10.1111/jce.15061 (PMC8360010; doi:10.1111/jce.15061)

**Supplement**

**Figure S1 – MARVEL 2 Algorithm**

The mechanical atrial sensing algorithm incorporates a post-ventricular blanking period and a dual threshold detection method. The first threshold (A3 threshold) that occurs early is used for detecting the atrial contraction when A4 (atrial kick) occurs during the A3 (passive filling) time and is less sensitive. The second threshold (A4 threshold) occurs later in the cycle after A3 has occurred and is more sensitive. A telemetry marker (VE) is displayed at the end of the A3 window.

**
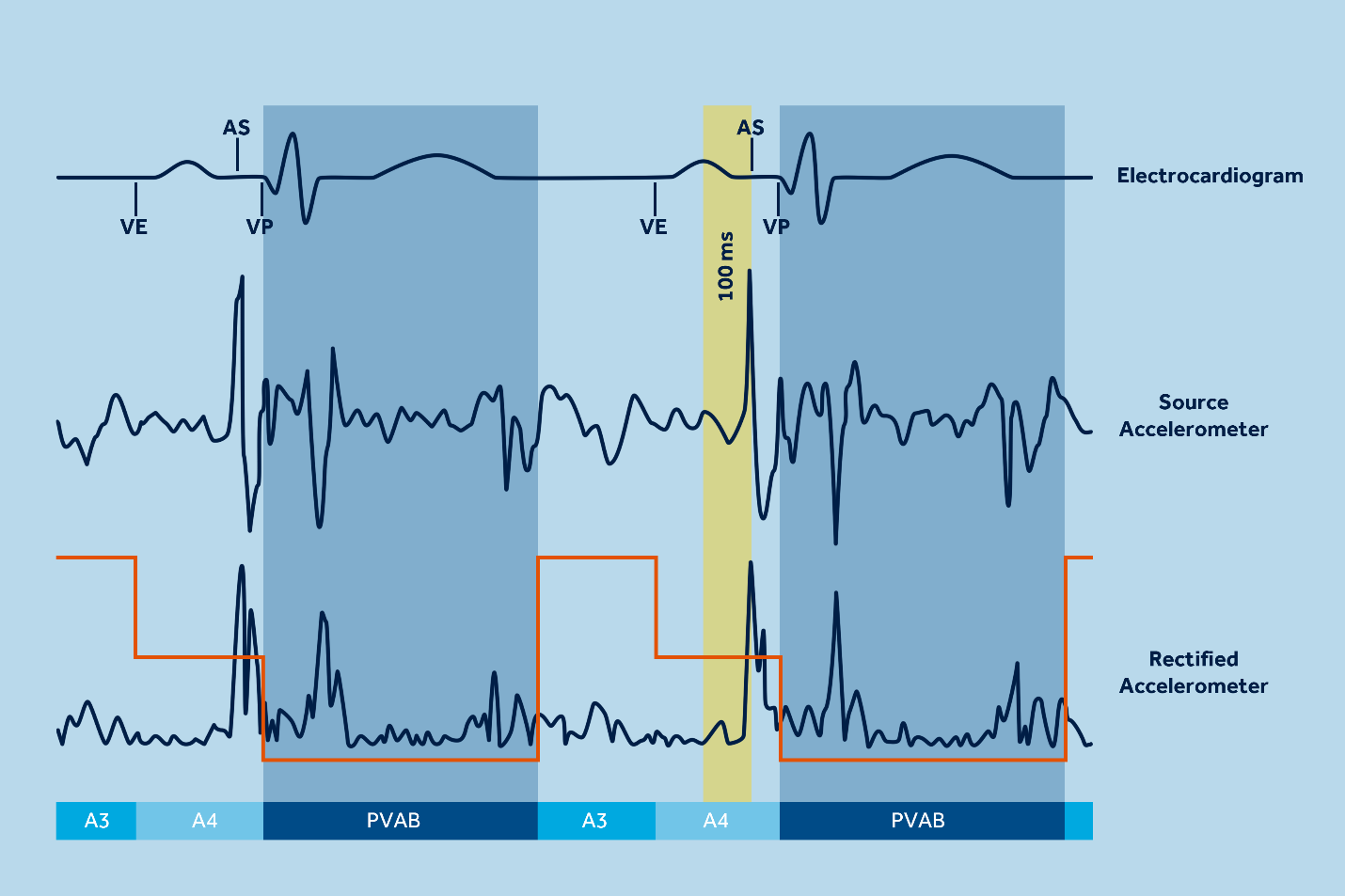
**

**(Image from: Steinwender CS, et al. J Am Coll Cardiol EP 2020;6:94-106).**

**Figure S2 – Idioventricular Rhythm with AV Conduction mode Switch On and then Off**

*Ventricular escape rhythms above 40 bpm with AV conduction mode switch on will mode switch to VVI-40 (left of MS line). AV conduction mode switch turned off at MS line to provide AV synchronous pacing. AS: Atrial sense; VP: ventricular pace; VE: end of A3 (ventricular) window.*


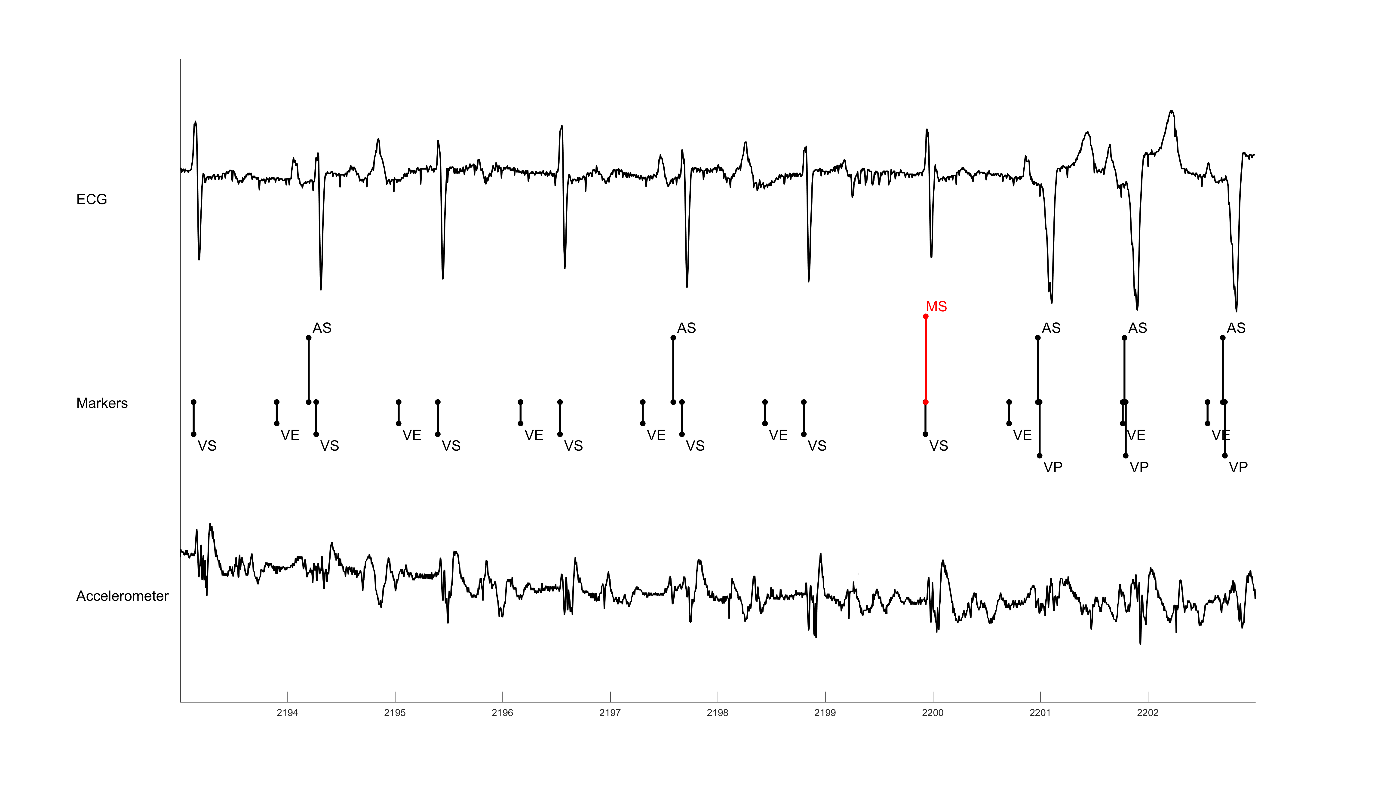


**Figure S3 – Behaviors during AF**

**S3A – Non conducting AF with no atrial senses**

*Atrial fibrillation without intrinsic conduction and without atrial sensing.*


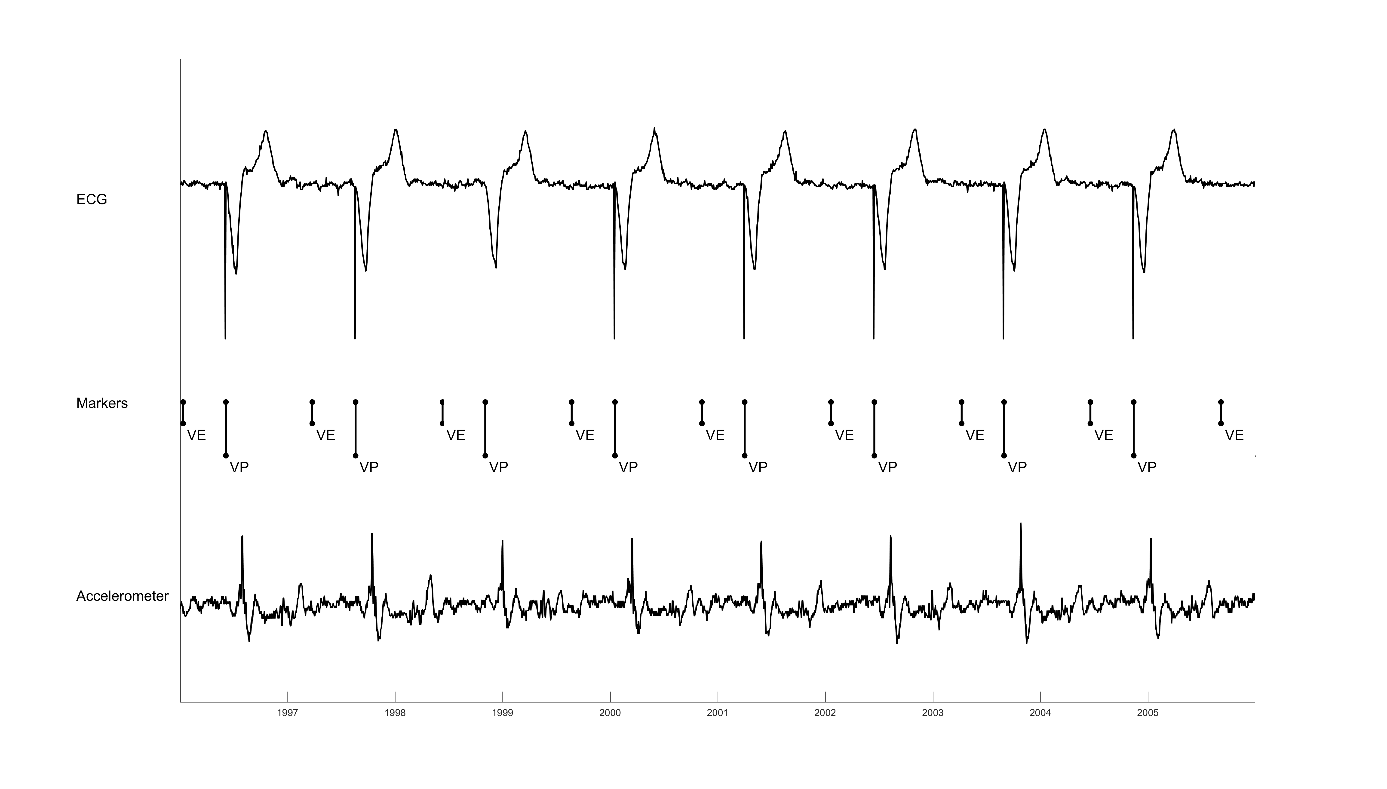


**S3B – Non conducting AF with intermittent atrial sensing**

*Atrial fibrillation without intrinsic conduction with intermittent atrial oversensing (AS).*


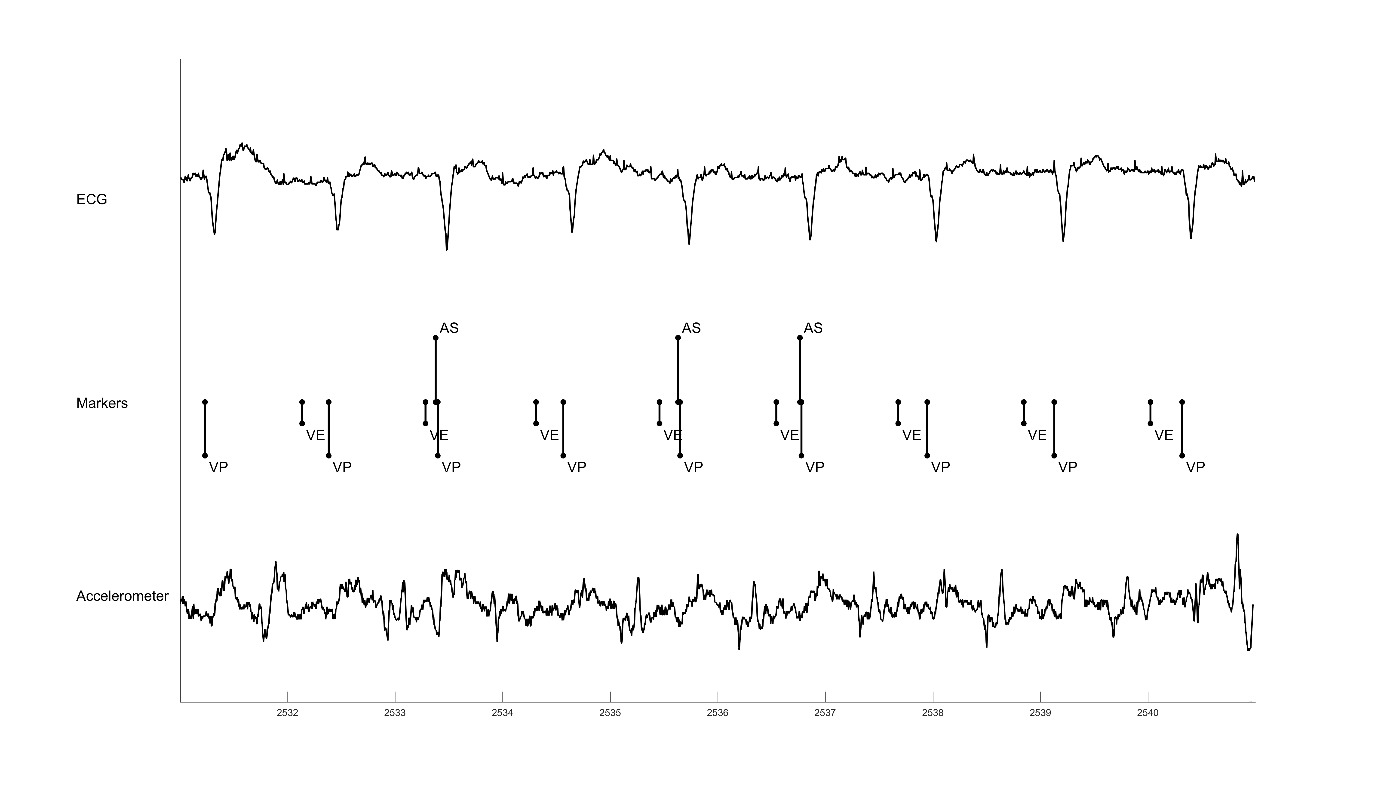


**S3C – Conducting AF with mode switch to VVI-40**

*Atrial fibrillation with intrinsic rhythm and activation of the mode switch (MS) as intrinsic ventricular rhythm >40 bpm.*


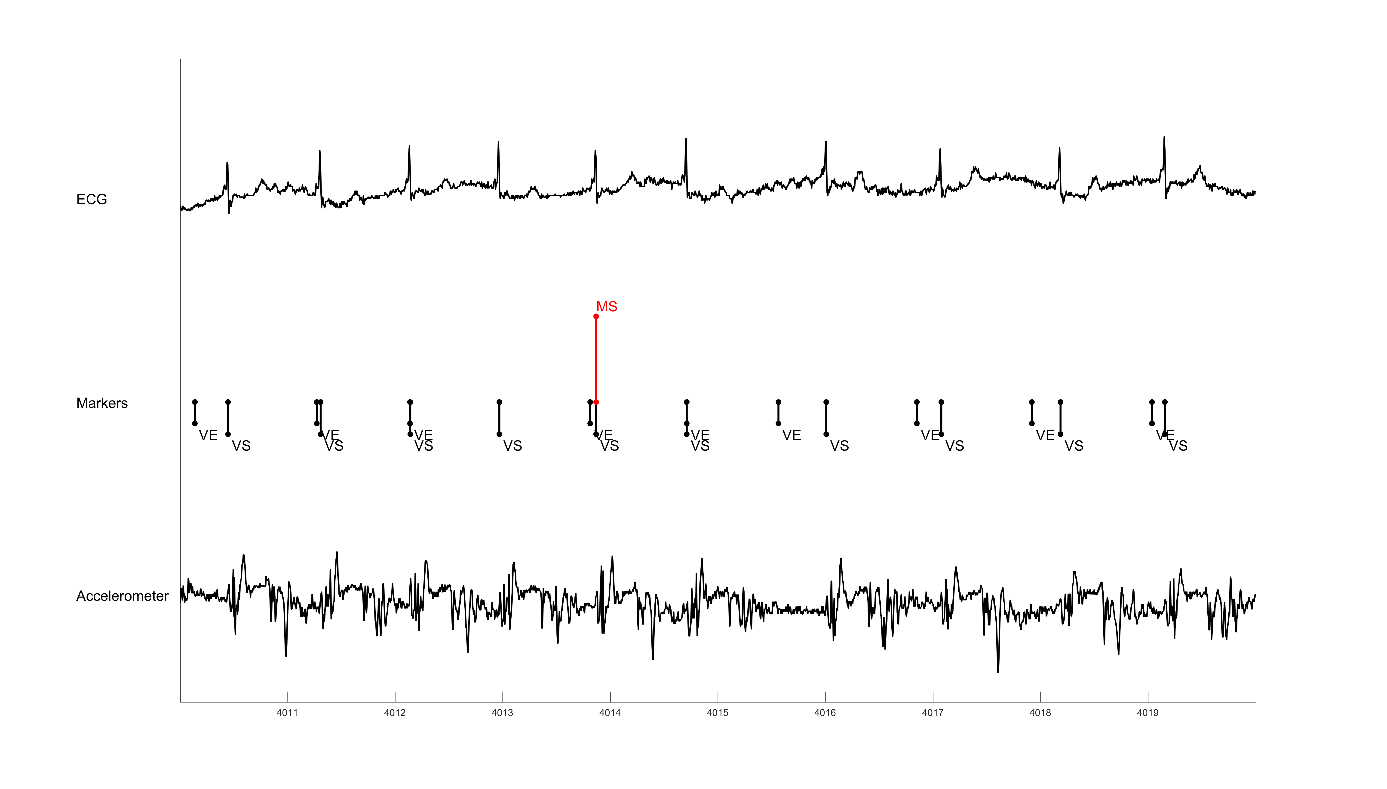


**Figure S4A – Rate smoothing pacing prior to P-wave**

*During sinus arrhythmia, short rate smoothing intervals may prematurely pace the ventricles during long P-P cycles (arrow). Increasing the rate smoothing delta may improve AV synchrony in this case.*


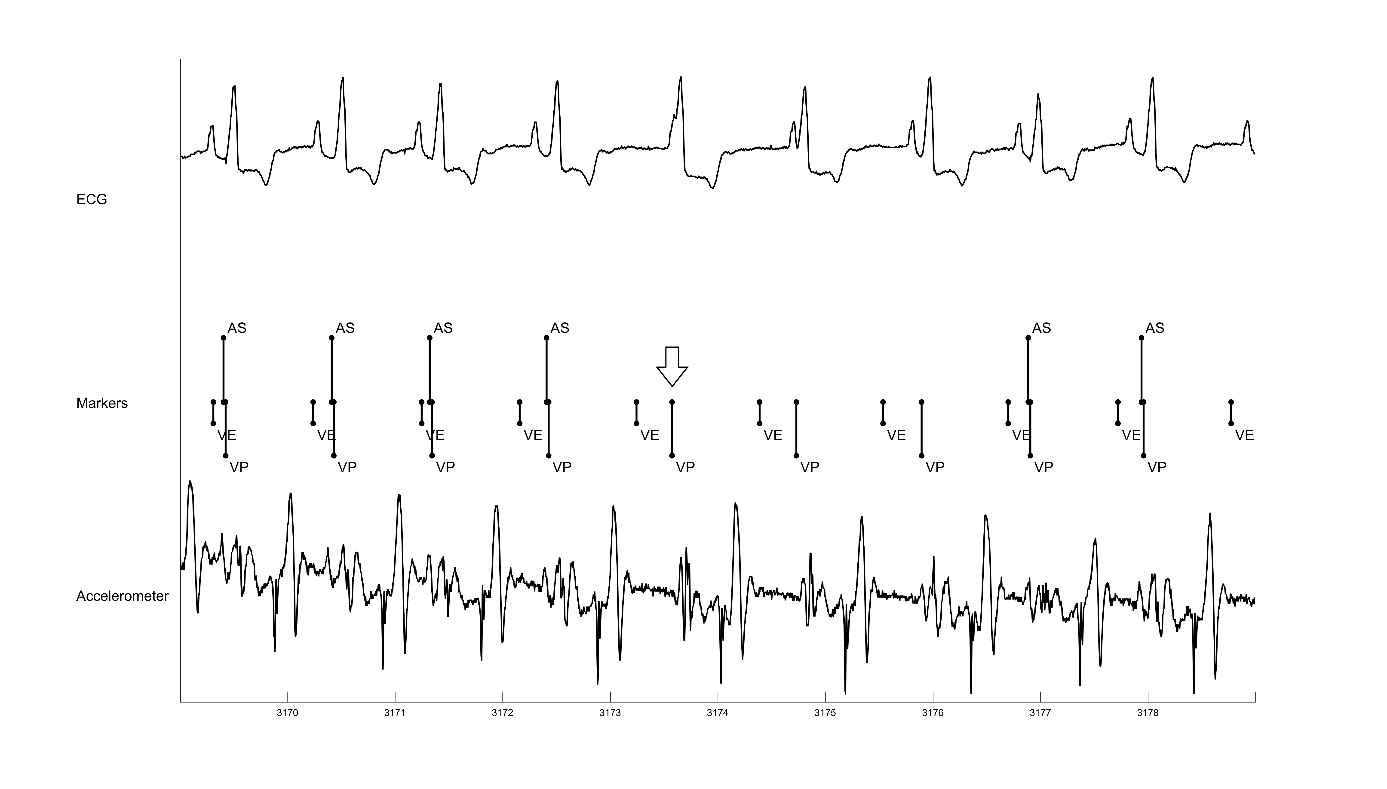


**Figure S4B – Rate smoothing delta too long leading to loss of AV synchrony**

*At higher sinus rates, long rate smoothing intervals may pace the ventricles (arrow) with P-wave occurring during ventricular systole or blanking. Decreasing the rate smoothing delta may improve AV synchrony in this case.*


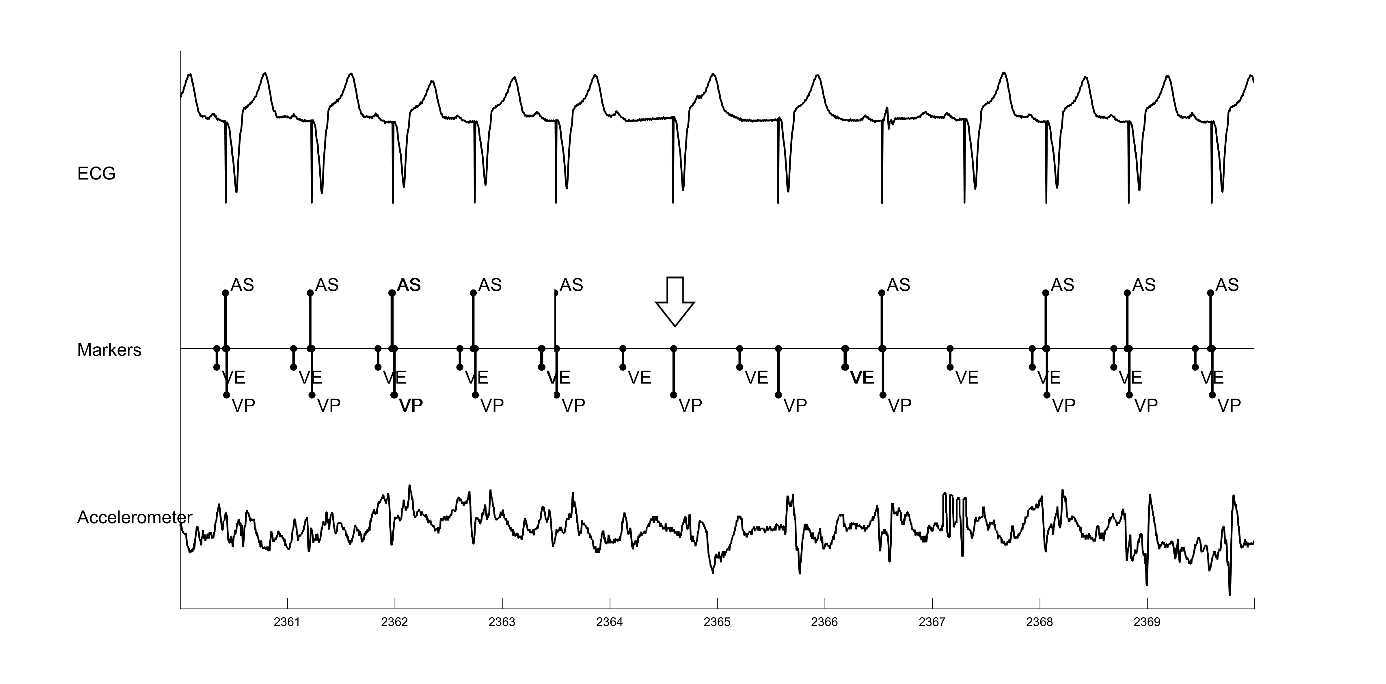


**Figure S4C – Rate smoothing delta decreased leading to improved AV synchrony**

*After rate smoothing delta was decreased in patient shown in Figure S3B, rate smoothing paces during intermittent undersensing (arrows) do not disrupt AV synchrony.*


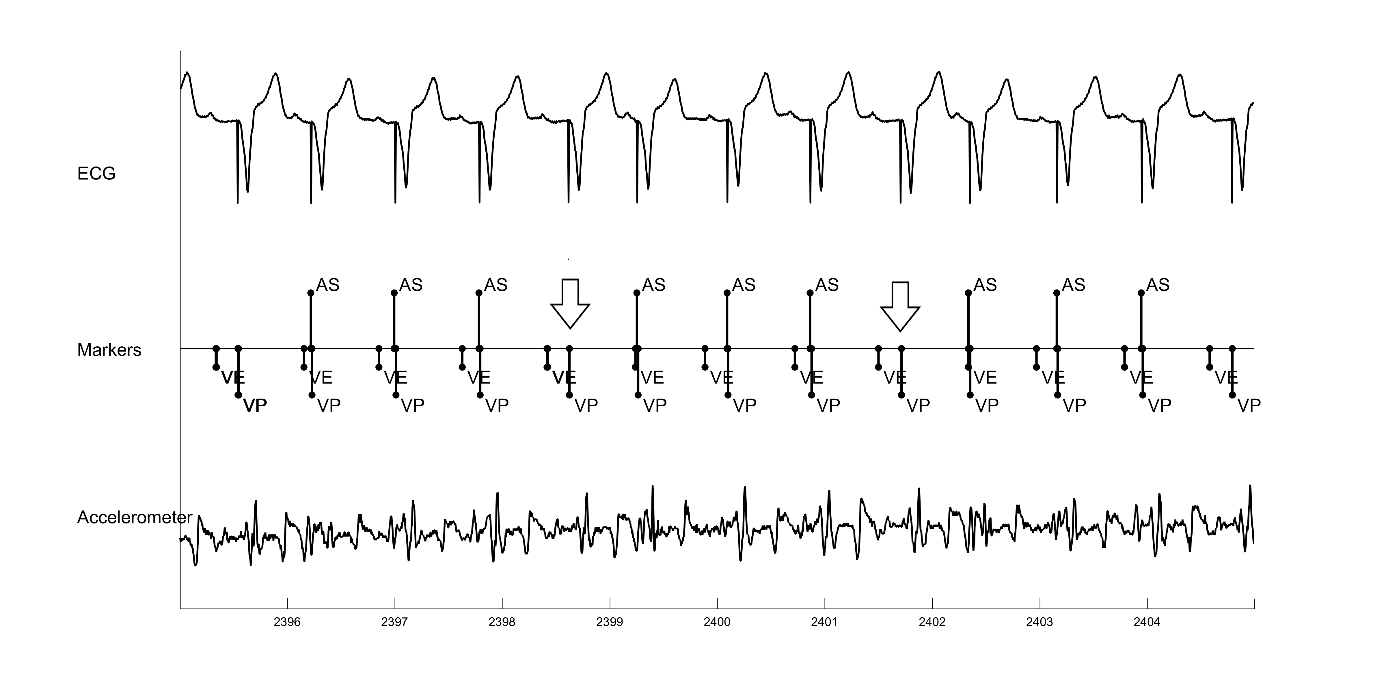

Supplement: Supplementary file 1 — Supporting information. [file JCE-32-1947-s001.docx]
